# Data-Driven Collaboration between Hospitals and Other Healthcare Organisations in Europe During the COVID-19 Pandemic: An Explanatory Sequential Mixed-Methods Study among Mid-Level Hospital Managers

**DOI:** 10.5334/ijic.6990

**Published:** 2023-06-16

**Authors:** Damir Ivankovic, Pascal Garel, Niek Klazinga, Dionne Kringos

**Affiliations:** 1 Amsterdam UMC location University of Amsterdam, Department of Public and Occupational Health, Meibergdreef 9, Amsterdam, The Netherlands; 2 Amsterdam Public Health research institute, Quality of Care, Amsterdam, The Netherlands; 3 The European Hospital and Healthcare Federation, Brussels, Belgium

**Keywords:** COVID-19, hospitals, mid-level hospital management, data-driven collaboration, integration

## Abstract

**Introduction:**

Data and digital infrastructure drive collaboration and help develop integrated healthcare systems and services. COVID-19 induced changes to collaboration between healthcare organisations, which previously often happened in fragmented and competitive ways. New collaborative practices relied on data and were crucial in managing coordinated responses to the pandemic. In this study, we explored data-driven collaboration between European hospitals and other healthcare organisations in 2021 by identifying common themes, lessons learned and implications going forward.

**Methods:**

Study participants were recruited from an existing Europe-wide community of mid-level hospital managers. For data collection, we ran an online survey, conducted multi-case study interviews and organised webinars. Data were analysed using descriptive statistics, thematic analysis and cross-case synthesis.

**Results:**

Mid-level hospital managers from 18 European countries reported an increase in data exchange between healthcare organisations during the COVID-19 pandemic. Data-driven collaborative practices were goal-oriented and focused on the optimisation of hospitals’ governance functions, innovation in organisational models and improvements to data infrastructure. This was often made possible by temporarily overcoming system complexities, which would otherwise hinder collaboration and innovation. Sustainability of these developments remains a challenge.

**Discussion:**

Mid-level hospital managers form a huge potential of reacting and collaborating when needed, including rapidly setting up novel partnerships and redefining established processes. Major post-COVID unmet medical needs are linked to hospital care provision, including diagnostic and therapeutic backlogs. Tackling these will require rethinking of the position of hospitals within healthcare systems, including their role in care integration.

**Conclusion:**

Learning from COVID-19-induced developments in data-driven collaboration between hospitals and other healthcare organisations is important to address systemic barriers, sustain resilience and further build transformative capacity to help build better integrated healthcare systems.

## Introduction

Since 2020, the world has faced unprecedented challenges posed by the COVID-19 pandemic [1,2]. To counter negative effects on individual and population health, healthcare organisations and individuals participated in understanding, managing and communicating the crisis [3,4,5,6,7]. This included urgently tackling the direct threat of a novel infectious disease outbreak while at the same time dealing with major disruptions to the provision of routine health care services [8,9,10,11,12,13]. Notably, the pandemic caused significant diagnostic and therapeutic backlogs in the provision of hospital care [9,14,15]. The collaboration between hospitals and other healthcare organisations, historically often fragmented and competitive, was replaced with goal-oriented collaborative practices, relying heavily on data [16,17,18]. Consequently, the pandemic highlighted the need for health data that is relevant, accurate, timely, secure, inclusive, available, complete, linkable and comparable [19,20].

Beyond COVID-19, Europe is faced with ageing populations, health workforce shortages and increasingly unequal societies [21,22,23]. Tackling these challenges, in a sustainable manner, requires redesigning service delivery patterns and better integration of care provided by hospitals and other types of health care organisations. Data and digital infrastructure are hugely important in driving this transformation [24,25].

Data-driven collaboration in healthcare ranges from data-informed interpersonal communication to fully automated collection, analysis and exchange of data between organisations and systems [26,27,28]. Having and sharing data proved crucial for decision-making on all levels during the pandemic [29]. This included clinical records of COVID-19 patients but also data on acutely relevant resources, such as hospital capacity, staff, personal protective equipment and respirators. Majority of scientific and anecdotal accounts reported increased data sharing and collaboration between clinicians, clinical and public health healthcare organisations, governments and citizens, hospitals and long-term care organisations, and between the public and the private sector in healthcare [30,31,32,33,34,35,36]. However, a knowledge gap exists in understanding the extent, nature and sustainability of these practices from the perspective of mid-level hospital managers in Europe.

As a European platform of national hospital and healthcare associations, the European Hospital and Healthcare Federation (HOPE) has a long history of building and maintaining a community of hospital managers [37]. During the COVID-19 pandemic, this community has been on the front lines of tackling the crisis, making them highly relevant stakeholders in exploring data-driven collaboration between hospitals and other healthcare organisations.

In this study, we focused on the perspective of mid-level hospital managers around Europe in 2021, during the second year of the COVID-19 pandemic. We aimed to explore (i) what happened to data-driven collaboration between hospitals and other healthcare organisations, (ii) what were the common themes of these collaborative efforts and (iii) which lessons – for practice, policy and research – can be learnt, going forward?

## Research methods

### Study design, participants and definitions

We conducted an explanatory sequential mixed-methods study, using a survey, case studies and webinars [38]. Our core research team of healthcare performance intelligence researchers, worked in collaboration with the HOPE Secretariat. HOPE represents national hospital and healthcare organisations from 30 European countries and has, since 1981, organised a professional training scheme called the HOPE Exchange Programme [39]. Aimed at mid-level hospital managers, the Exchange annually involves around 150 participants and consists of a four-week visit to hospitals and other healthcare organisations in another, host country and ends with a joint conference. Due to the COVID-19 pandemic, the Exchange did not take place in 2020 and 2021.

2019 and 2022 Exchange participants, their local hosts and the wider HOPE community were involved in this study. Research steps included an online survey, semi-structured case-study interviews and webinars, all of which were used for data collection. The webinars were also used for data validation.

The research team previously worked with HOPE and the 2019 Exchange Programme participants on a study exploring managerial use of performance data in hospitals [40]. This implied joint understanding of relevant concepts, used in this study, such as data and public health institutions. For instance, study participants were aware that data inferred both individual, patient level data as well as operational-level performance data. Likewise, public health institutions were specifically understood as national, regional and local organisations that deal with disease and risk factor surveillance, epidemiologic investigation, public health research and response to public health emergencies [41].

### Survey

An online survey was conducted, eliciting information on data-driven collaboration between hospitals and other healthcare organisations across Europe in 2021. The inclusion criteria for participation were involvement in the 2019/2022 Exchange as a participant or local host and working in a hospital, which treated COVID-19 patients. Considering the increased workload on hospital staff during the pandemic, the survey was developed in a one-minute format, and was tested and administered in English, using Typeform™ [42]. Cognitive pretesting took place in November 2020 and involved six testers from five countries (Appendix 2: Survey cognitive testing informants), one of whom was a native-English speaker [43]. Pretesting prompted survey language edits to improve understandability for non-native speakers. The survey finally consisted of nine mandatory closed-ended questions and one optional open-ended question (Appendix 3: Online survey). Invitations were sent via email through the HOPE Secretariat. Participants were informed about the voluntary and anonymous nature of the study, its objectives and the intended dissemination of results through webinars and a scientific publication. The survey was launched on 12 January 2021, two reminders were sent, and the data collection ended on 16 February 2021.

### Multiple-case study

For the multiple-case study, we engaged in a series of semi-structured interviews, collecting cases of working with COVID-19 data in hospitals and exchanging data with other health care organisations. Survey results informed the design of the case study guide (Appendix 4: Case study interview invitation). Following invitations though the HOPE Secretariat and HOPE National Coordinators in April 2021, eight mid-level hospital managers accepted the invitation for an interview. Remote video interviews took place between 21 April and 22 June 2021 and were recorded following verbal approval by participants. All interviews were conducted by the first author in English, each lasted on average 30 minutes and the recordings were transcribed for the analysis.

For context, Table 1 provides an overview of recent, COVID-induced health data and governance developments in the eight European countries included in case studies, as reported by the OECD [44,45].

**Table 1:** Overview of COVID-induced developments in health information systems, in countries contributing to country cases, in this study. NA = Data not available. Two-letter country codes: NL = the Netherlands, FI = Finland, BE = Belgium, FR = France, IE = Ireland, MD = Moldova and PL = Poland.


		NL	FI	BE	FR	IE	MD	PL

**As a result of COVID-19, country has introduced:**	New technologies to improve health data availability, accessibility, sharing or data privacy and security protections	No	No	Yes	No	No	NA	Yes

	Legal, regulatory or policy reforms to improve health data availability, accessibility, or sharing	No	No	Yes	No	No	NA	Yes

	Legal, regulatory or policy reforms to improve health data privacy or security protections	Yes	No	Yes	No	No	NA	Yes

	Financial incentives to improve health data availability, accessibility, sharing or data privacy and security protection	No	No	Yes	No	No	NA	No

**As a result of COVID-19, improvements to key national personal health datasets were made in terms of:**	Timeliness	Yes	No	Yes	No	No	NA	Yes

	Quality, coverage, and/or completeness	Yes	No	Yes	No	No	NA	Yes


### Webinars

The HOPE Secretariat and the research team jointly organised two webinars to engage with stakeholders and to present, validate and discuss survey and case study findings. HOPE National Coordinators, local Exchange hosts and Exchange Programme participants were invited. The first webinar took place on 26 February 2021 for one hour and involved 45 participants. Research team presented the survey results and moderated a panel and audience discussion, involving cognitive testing informants (n = 4). The second webinar was organised on 11 June 2021, lasted 90 minutes and involved 35 participants. Research team presented four case studies, collected prior to the webinar, and moderated another panel and audience discussion, involving case study informants. Both webinars were recorded in agreement with participants and made publicly available through HOPE’s YouTube channel [46,47].

### Data analysis and research design assessment

Survey data were analysed in Excel using descriptive univariate statistics, for closed-ended questions and thematic analysis, for the open-ended question [48,49]. Interview and webinar transcripts were used for case study analysis, employing the cross-case synthesis methodology and deductively identifying thematic patterns, again using Excel [50]. Starting with theoretical proposition, based on initial survey results, the analytical aim was to maintain case integrity and contrast and compare any patterns across the cases, presenting them as lessons learnt and future implications.

Key validity aspects of case study research design were accounted for and continually assessed: (i) construct validity, by triangulating survey, interview and webinar data and also by reviewing key informants case study reports during webinars; (ii) internal validity, through clear research framework development and pattern matching with survey findings; (iii) external validity, by employing the analytic generalisation approach and defined criteria for the case study selection and (iv) reliability, by developing and publishing the case study guide (Appendix 4: Case study interview invitation) [50].

Data analysis was conducted by the first and reviewed by co-authors. Close engagement with relevant stakeholders, through webinars, was also used for dissemination and validation of the survey and interview results. The study methodology adhered to the Consolidated Criteria for Reporting Qualitative Research (Appendix 1: COREQ Checklist) [51].

### Ethical approval

The research protocol was developed in accordance with the ethical requirements of the primary research affiliation to Amsterdam University Medical Centers of the University of Amsterdam. Participants provided verbal consent at the start of webinars and interviews. Confidentiality was assured by removing identifying information throughout the paper. One participant withdrew their consent after having participated in a case study interview, due to unclear organisational policies. Those data were deleted, and the results were not used.

## Results

### Participants

Approximately 250 healthcare managers were invited to participate and one-third completed the survey (86/250; 34.4%). Respondents that replied not being affiliated to hospitals treating COVID-19 patients were excluded from further analysis. The remaining 62 full replies, from respondents working in 18 European countries, mostly came from Poland (10/62; 16.1%), the Netherlands (9/62; 14.5%) and Austria (6/62; 9.7%). Respondents were usually affiliated to larger, regional or teaching (25/62; 40.3%) and university hospitals (24/62; 38.7%). For subsequent research steps, eight key informants, from seven different countries, contributed to case study interviews (Appendix 5: Case study interview informants). A total of 80 participants joined the two webinars.

### Survey study: Data-driven collaboration during COVID-19

When asked about the best metaphor for their organisation’s COVID-19 data exchange with other hospitals and healthcare organisations, around a half of the survey respondents compared their affiliated hospital to “an island connected by the bridge” (29/62; 46.8%) and half to “an island connected by a ferry boat” (29/62, 46.8%). Only a few opted for “an isolated island” metaphor (4/62; 6.4%).

Three-quarter of respondents reported in increase in collaboration between their affiliated hospitals and other healthcare organisations during the COVID-19 pandemic (scores = 4 or 5; 46/62; 74.2%). Recoded Likert-scale response score average was 3.98 (N = 62) along a gradient from “collaboration decreased” (score = 1), through “it stayed the same” (score = 3) to “collaboration increased” (score = 5).

**Figure 1:**
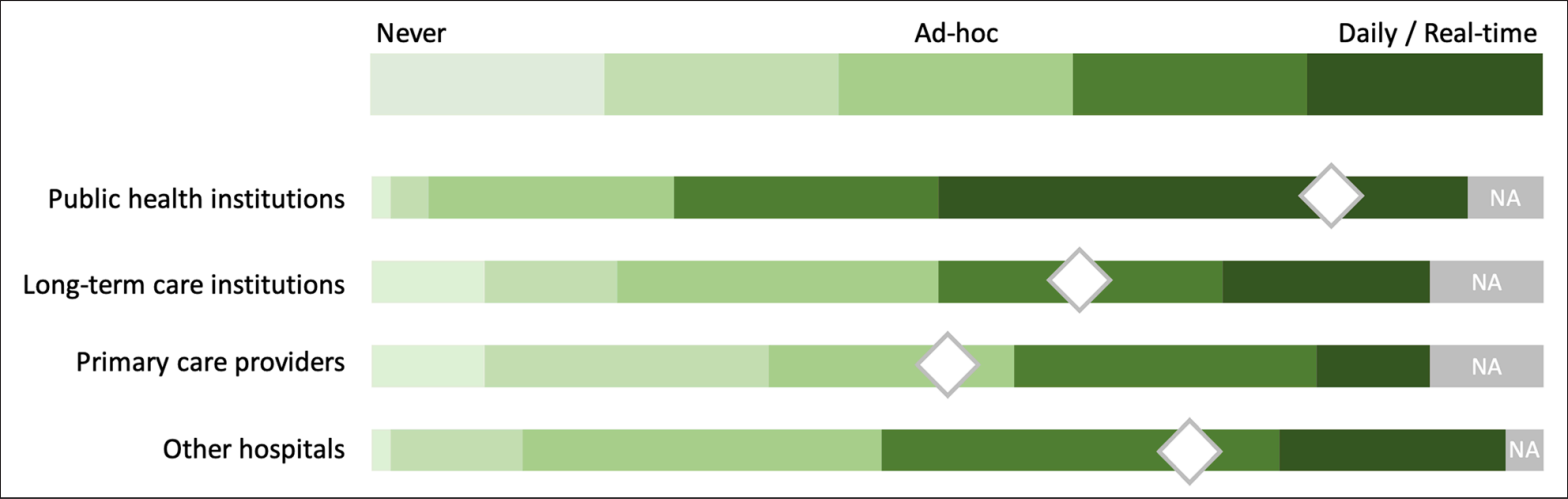
“To what extent does your hospital exchange COVID-related data with the following organisations and their data systems?” Perceived level/intensity of COVID-19 data exchange between respondents’ hospitals and other healthcare organisations (N = 62). Note: Diamond shapes represent recoded Likert-scale responses averages (from 1 = “Never”, through 3 = “Ad-hoc” to 5 = “Daily/real-time”). NA = Not applicable.

The extent of COVID-related data exchange between respondents’ affiliated hospitals and other healthcare organisations was also assessed. Data exchange with public health institutions was on average close to “real-time”, while it mostly happened “ad-hoc” with other hospitals, long-term care institutions and primary care providers (Figure 1).

When elaborating on lessons learnt with the exchange of COVID-19 related data, free-text replies could be grouped into “positive”, “negative” and “neutral” sentiments, as presented in Table 2 below.

**Table 2:** “Please elaborate on lessons learnt with the exchange of COVID-19 related data”. Free-text replies grouped following thematic analysis (N = 30).


SENTIMENT	LESSONS LEARNT

Positive	Pandemic as an opportunity for more data-driven interaction and collaboration. COVID-19 testing and vaccination as a test for existing data systems. Improvements in semantic and technical aspects of data exchange. Improved links to other care levels and organisations.Improved links within organisations themselves.

Neutral	Nothing changed. Still a lot to learn.

Negative	Siloed data systems and governance. One-directional sharing of data. Data production increased but in a disorganised way.


### Multiple-case study: Common themes of collaborative practices

A number of common themes were identified through the case study analysis. Improvement and innovation initiatives dealt with streamlining governance. Hospitals around Europe established new and strengthened existing partnerships. This often required re-engineering care provision models and improving data infrastructure.

Engaging in, more or less formalised, public-private partnerships (PPP) based on data sharing, was often mentioned, as a form of COVID-19-induced collaboration for hospitals around Europe.

In a case study from Belgium, an existing network of five major Brussels-based public and academic hospitals invited the two largest private hospitals in the area to join the network.

“The discussion to add private hospitals to the network started already pre-COVID but when COVID-19 struck, the decision was made in two days. Private hospitals accepted the invitation, and the collaboration started by holding weekly meetings of medical directors of all seven hospitals. The collaboration was supported by the newly developed data dashboard, used by all participating hospitals, with real-time information on bed capacity as well as protective and therapeutical equipment capacity. This was later expanded with the ability to share patient summary health records. Data needed for the dashboards was promptly defined, collected and shared within the network. It was all done very fast, so data quality was bad in the beginning, especially differences in data definitions across data sets. It improved over the first few months of the pandemic, though. This shared data were widely used, especially for managing and facilitating patient transfers between hospitals. It worked within the region, across Belgium but also with other countries, such as Germany and the Netherlands” (Key informant #3).

Safety net agreement in Ireland, between the Health Service Executive and multiple private hospitals, was yet another example [52]. This enabled provision of surge capacity care to patients in private hospitals during the pandemic’s peaks and dealing with backlog in diagnostic and therapeutic procedures, following peaks. Prior to the pandemic, public and private hospitals in Ireland shared little to no data between each other. To be able to link data across organisations, a nation-wide temporary unique patient identifier was introduced.

“Administrators in the public hospital would first assign this number to a patient and then communicate it to the private hospital. Private hospitals then logged everything under this code. This worked well but was very manual. Input, print, scan, email…” (Key informant #5).

Similar PPP arrangements were put in place in France, with the private sector taking over some of the recovering COVID-19 patients from public hospitals, to relieve the pressure on intensive care bed capacity and staff there.

“This was all facilitated by the newly developed data system, mostly focused on capacity management, and coordinated on the national level at the Ministry of Health. It worked surprisingly well and fast” (Key informant #4).

Patient transfers among hospitals and other healthcare organisations, supported by rapid advancements in data infrastructure, were also a common theme. For example, in the Netherlands, starting in Amsterdam and then expanding to the whole North-Holland province, hospitals and long-term care (LTC) organisations upscaled the use of existing data systems, and introduced new ones, to exchange information on geriatric patients [53]. Besides being able to transparently see and manage hospital and LTC bed capacity real-time, these newly introduced systems also enabled the exchange of doctor and nurse letters and soon after full patient electronic health records among organisations.

“Collaboration increased substantially between hospitals themselves and with LTC but also with insurance companies. Starting with hospital to LTC communication, these new data solutions were not only used for LTC capacity planning and patient data transfer but also for other COVID-19 intensive care patients and the fair share agreement. Because of COVID-19, everybody knows so much more about each other these days” (Key informant #1).

Most cases mentioned changes and improvements to care provision that went beyond collaboration for capacity management and patient transfers. In many cases, care provision also significantly shifted outside of hospital walls, often aided by a more widespread use of telehealth services. Informants emphasised the importance of available and interoperable patient data for this kind of collaboration. Additionally, reimbursement schemes for teleconsultation services proved crucial in the increased uptake from the providers’ side.

During the pandemic, healthcare workforce demand exceeded supply, with data collection burden increasing at the same time. To partly tackle this issue, the Polish Armed Forces provided support to civilian healthcare services in Poland. Soldiers were deployed to all public hospitals where they assisted, among other activities, in collecting data on bed and protective equipment capacity multiple times a day and communicating those to the regional coordinators from the Ministry of Health.

“It was kind of strange to see soldiers roaming around hospitals, but they did help us quite a lot, allowing us to focus more on patients, and we are grateful for that” (Key informant #8).

### Lessons learnt

Informants were encouraged to summarise lessons learnt and their predictions for the future of data-driven collaboration and sustainability of changes introduced during the pandemic.

First lesson was that allowing individuals and organisations to temporarily overcome or bypass usual system complexities helped deal with the crisis more effectively. This, among others, included establishing interorganisational, and often public-private, partnerships and collaboration. However, sustainability of these efforts remained questionable. An informant commented:

“Firstly, this emergency thinking needs to stop. Sure, some longer lasting improvements will come out of this situation and stay but new challenges will also pop up, such as the financial sustainability of the system, which was mostly ignored during COVID-19. Also, catching-up on delayed care and getting patients back to hospitals, for instance” (Key informant #3).

Next lesson was that the introduction or upscaling of innovative care provision modalities, such as the use of telehealth services, helped manage the pandemic better. Again, most informants agreed that the continued use of such care provision modalities will require introduction of more permanent incentivising reimbursement systems.

“Hospitals in this country are usually competing with each other. Right now, it is ‘we’ but I fear we will soon go back to ‘us’… mostly because of volume-based contracting agreements” (Key informant #1).

Finally, the pandemic also challenged ongoing or planned health system reforms in some European countries. The lesson here was that operationally and strategically managing and improving health systems in “normal” times still must account for resilience in absorbing and recovering from disruptions, such as a pandemic, and be able to learn and adapt for the future.

“We realised how much we lacked linkable data so this might be an opportunity for the future to work on digitalisation and use private sector’s experience in e-health and data-driven work to help the public sector out” (Key informant #6).

“Following COVID-19 and PPP experiences, the public sector might need to re-think the ongoing reform process and start using the private sector more. For instance, to clear the backlog of patients in the public sector, but in a ‘smart’ way, potentially using a bidding system of some sort” (Key informant #5).

## Discussion

With this study, we aimed to investigate data-driven collaboration that took place between hospitals and other healthcare organisations in Europe in 2021, during the second year of the COVID-19 pandemic. We did so by documenting and analysing first hand experiences of mid-level hospital managers, taking stock of recent developments and to identifying common themes and lessons learnt from these collaborative practices.

Our research previously focused on middle management in hospitals and their use of data for operational- and strategic-level decision-making [40]. Building on that work, in the context of the COVID-19 pandemic and considering the growing relevance and need for integrated care systems and services, we expanded our scope to look at inter-organisational collaborative practices. Collaboration presents a necessary element for establishing linkages and coordination, in the continuum leading to fully integrated care [54]. Many mutually reinforcing, structural and relational, factors drive collaboration in healthcare. These, among others, include governance, shared goals, trust and information exchange [55].

The pandemic introduced an unprecedented level of importance and urgency to act [56,57]. Mid-level hospital managers, participating in our survey, almost never perceived their hospitals as “isolated islands” and generally reported an increase in data exchange between hospitals and other healthcare organisations in 2021. This was especially true for exchanging data with public health institutions, which is unsurprising, considering an ongoing pandemic. We also identified that data-driven collaborative practices were highly goal-oriented and focused on improvements to hospitals’ governance functions, organisational models and data infrastructure. In practice, this usually meant re-designing care provision, establishing new partnerships and optimising data interoperability within and across healthcare organisations. Findings also signalled that a key enabling factor might have been the ability to temporarily overcome system complexities, which would otherwise hinder collaboration and innovation. Most cases mentioned establishing, previously hard-to-implement, private-public partnerships as well as the upscaling of the – previously legally or financially limited – use of telehealth services.

On the governance side, collaborative developments included re-thinking existing competitive economical models, regionalisation and central planning efforts, new partnerships and improved transparency on capacity and outcomes. These were often based on data ecosystem communication, enabling further analytics-based insights and allowing for governance emphasising clear roles, focus on health outcomes, resilient structures and transparent processes [58]. Re-design of collaborative hospital organisational models mostly took place through innovation in care provision. A common approach was the substantially increased availability and uptake of telehealth services, taking place outside hospital walls and involving collaboration with other types and levels of healthcare services and payers [59,60,61]. With multiple factors causing almost universal shortage of relevant health workforce, another major collaboration theme was the health workforce task shifting, within and between organisations [62,63]. To secure that the system-level adaptive, absorptive and transformative workforce capacities are used optimally, collaboration focused on staff redeployment, according to needs, and changes to recruitment, onboarding, and training processes [64,65]. The needed level of situational awareness, achieved through having access to quality data, was crucial during the pandemic. Despite many healthcare systems being data-rich, a robust public health reporting infrastructure, offering actionable and interoperable data to act during a pandemic, remained a challenge [66]. To additionally support these efforts, a more collaborative and transparent approach to reporting and managing hospitals’ bed and equipment capacity, was taken. For instance, the use of data dashboards, as a collaborative, managerial and public reporting tool, has substantially increased worldwide during the COVID-10 pandemic and, the research suggests, might stay, post-COVID-19 [67,68,69].

Not all developments were perceived positively. For instance, “unorganised increase in data production” and its “one-directional sharing” were recognised as some of the shortcomings. Lack of formative feedback, which should be actionable for clinical and organisational quality improvement work, is a well-researched issue in healthcare management and integration [70,71,72]. So are the suboptimal governance approaches and the lack of strategic planning, related to performance data on both national and organisational levels [73,74]. Considering the existing academic and policy focus on data- and analytics-based insights feeding into decision-making as well as on the evolving role of hospitals in the broader healthcare system context, majority of these developments are not completely new [75]. In fact, many conceptually and practically predate the COVID-19 pandemic and have also been elucidated and discussed in our previous work with the same community of healthcare managers [76,40].

Finally, our findings signalled that, already by mid-2021, introducing new and maintaining existing collaborations between hospitals and other organisations posed a challenge. Sustainability of these developments and the long-term strategic planning of collaborative data governance should benefit from Europe-wide mechanisms such as the Recovery and Resilience Facility funds and the European Health Data Space initiative [77].

Discussing integrated clinical care of COVID-19 patients during the pandemic was beyond the scope of this study [78,79]. However, we hope to have elucidated some common models and features of data-driven collaboration between hospitals and other healthcare organisations, which occurred during the pandemic. In turn, these might help effectively tackle future key system pressures, such as workforce shortages and long waiting lists for elective procedures. More importantly, as patients – including those with post-COVID conditions – cross care boundaries in the future, these findings might signal specific approaches where data-driven collaboration could lend itself to drive integration.

This work benefited from working with an established community of mid-level hospital managers across Europe. We used sequential, mixed-methods survey and case study research methodology and involved research subjects iteratively throughout all research phases. This methodological approach is recommended when boundaries between the context – the COVID-19 pandemic – and the phenomenon researched – data-driven collaborative practices – are less than clear [50]. It also allowed us to address a relatively broad research question. However, this research approach is also resource-demanding and calls for substantial investment of time and effort from researchers and considerable motivation and involvement from informants. The latter was especially challenging, considering the workload and fatigue, which hospital managers experienced during the COVID-19 pandemic. Further limitation of this work is the fact that the research results are limited to the HOPE Exchange Programme participants and are not generalisable to healthcare managers elsewhere. Due to the purposive sampling and the limited pool of cases in this study, it was impossible to conduct comparisons between, or within, European countries. Finally, data privacy considerations and the resulting lack of demographic information on participants did not allow for a detailed analysis of non-responders.

## Conclusion

The COVID-19 pandemic had a major impact on the development of healthcare systems and health care services during the previous two and a half years. It facilitated new innovative collaborative models, with professional healthcare communities playing an important role in the process. Our results signal extensive and increasing data-driven collaboration between hospitals and other healthcare organisations in tackling the current pandemic and its indirect consequences. With the majority of unmet medical needs currently being linked to hospital care, these will become even more important in the near future [80]. Data systems and, more importantly – people behind data systems, have shown a huge potential of innovating and collaborating when needed. Besides examining which of these efforts achieved their goal in the short-term, sustainability also remains a challenge. Isolation helped curb the spread of the virus [81,82] but it was collaboration that enabled healthcare systems to adapt and absorb this major disruption. Our work signals that this transformative capacity can only be sustained if systemic barriers to meaningful collaboration are understood and managed.

## Data Accessibility Statement

All data relevant to the study are included in the article or uploaded as supplementary information. The data generated and/or analysed in the study are not publicly available due to participant anonymity.

## Additional Files

The additional files for this article can be found as follows:

10.5334/ijic.6990.s1 Appendix 1. Consolidated Criteria for Reporting Qualitative Research (COREQ) Checklist.

10.5334/ijic.6990.s2 Appendix 2. Survey cognitive testing informants.

10.5334/ijic.6990.s3 Appendix 3. Online survey (mobile version).

10.5334/ijic.6990.s4 Appendix 4. Case study interview invitation.

10.5334/ijic.6990.s5 Appendix 5. Case study interview informants.

## Author Contribuctions

DI, PG, DK and NK designed the study. All authors contributed to the development of the survey and interview guide, used in this study. DI prepared the online survey; PG administered it and DI initially analysed survey data. All authors reviewed and validated final survey results. PG and DI coordinated interview recruitment. DI conducted interviews, transcribed and initially analysed interview data. All authors analysed interview data. All authors participated in planning, preparation and execution of webinars. DI prepared the manuscript. All authors reviewed drafts of the manuscript and approved the final version.
