# Supplementary material for: Data-Driven Collaboration between Hospitals and Other Healthcare Organisations in Europe During the COVID-19 Pandemic: An Explanatory Sequential Mixed-Methods Study among Mid-Level Hospital Managers
Source: Int J Integr Care. 2023 Jun 16;23(2):28. doi: 10.5334/ijic.6990 (PMC10275210; doi:10.5334/ijic.6990)
Supplement: Appendix 1. — Consolidated Criteria for Reporting Qualitative Research (COREQ) Checklist. [file ijic-23-2-6990-s1.pdf]

**Appendix 1:** Consolidated Criteria for Reporting Qualitative Research (COREQ) Checklist[a]

| # | Item | Question/description | Answer | Section |
|---|---|---|---|---|
| **Domain 1: Research team and reflexivity** | | | | |
| **Personal Characteristics** | | | | |
| 1 | Interviewer/ facilitator | Which author/s conducted the interview or focus group? | DI | Methods |
| 2 | Credentials | What were the researcher's credentials? | DI: MD, MBA*<br>PG: MSc<br>DK: MSc, PhD<br>NK: MD, PhD<br>*PhD student | Methods and authors' list |
| 3 | Occupation | What was their occupation at the time of the study? | Researchers were members of an existing international network of healthcare performance intelligence professionals (HealthPros) or involved with the secretariat work of HOPE. | Methods |
| 4 | Gender | Was the researcher male or female? | One woman<br>Three men | Methods |
| 5 | Experience and training | What experience or training did the researcher have? | The research team included healthcare performance intelligence researchers and practitioners with previous experience of working with health data and policy, designing health system performance assessment frameworks and conducting health information system assessments in the European and global context | Methods |
| **Relationship with participants** | | | | |
| 6 | Relationship established | Was a relationship established prior to study commencement? | Informants were previous or future participants to the HOPE Exchange Programme. Throughout the study, informants were provided with study overviews and detailed survey/interview/webinar briefs. | Methods |
| 7 | Participant knowledge of the interviewer | What did the participants know about the researcher? e.g. personal goals, reasons for doing the research. | Informants all received a written overview of the study aims and objectives in English. The reasons for conducting the research were restated at the start of all interviews and webinars. | Methods |

| # | Item | Question/description | Answer | Section |
|---|---|---|---|---|
| 8 | Interviewer characteristics | What characteristics were reported about the interviewer/facilitator? e.g. Bias, assumptions, reasons and interests in the research topic | At the outset of each interview, the interviewer present introduced themselves by their areas of research and affiliations. | Methods |
| **Domain 2: study design** | | | | |
| **Theoretical framework** | | | | |
| 9 | Methodological orientation and Theory | What methodological orientation was stated to underpin the study? e.g. grounded theory, discourse analysis, ethnography, phenomenology, content analysis | An explanatory sequential mixed-methods, involving online survey, case study interviews and webinars were employed. Data collected through the online survey were analysed using univariate statistics, and thematic analysis for the final, open-ended question. These results sequentially informed the design and focus of country case semi-structured interviews. Interview transcripts were analysed using narrative analysis. Data analysis was conducted by the first author (DI) and reviewed by co-authors (PG, NK, DK). Close engagement with relevant stakeholders, through webinars, was also used for dissemination and validation of the survey and interview results. | Methods |
| **Participant selection** | | | | |
| 10 | Sampling | How were participants selected? e.g. purposive, convenience, consecutive, snowball | Purposive sampling based on target profile of participants working in mid-managerial positions in hospitals, which treated COVID-19 patients at the time of conducting the study, in HOPE member countries. | Methods |
| 11 | Method of approach | How were participants approached? e.g. face-to-face, telephone, mail, email | Multiple methods via email, including direct contacts and advice of local experts (national coordinators for HOPE). | Methods |
| 12 | Sample size | How many participants were in the study? | Eighty-six informants participated in the online survey. Eight participants joined the semi-structured case study interviews. Forty-five and fifty-five participants joined the February | Methods and Results |

| # | Item | Question/description | Answer | Section |
|---|---|---|---|---|
| | | | and June webinars (respectively). | |
| 13 | Non-participation | How many people refused to participate or dropped out? Reasons? | Out of 86 respondents to the online survey, 24 were not affiliated with hospitals treating COVID-19 patients at the time of the survey. This data was excluded from the analysis of survey results.<br>One participant withdrew their consent after having participated in a country case semi-structured interview, due to unclear organisational policies. This data was deleted, and results were not used for this work. | Methods and Results |
| **Setting** | | | | |
| 14 | Setting of data collection | Where was the data collected? e.g. home, clinic, workplace | Through an online survey, online interviews (n=8) and webinars (n=2). | Methods |
| 15 | Presence of non-participants | Was anyone else present besides the participants and researchers | For the interviews – no. For the webinars – yes, broader HOPE community members were also invited for webinars. | Methods |
| 16 | Description of sample | What are the important characteristics of the sample? e.g. demographic data, date | Full online survey replies, included in the analysis (n=62), came from respondents working in 18 European countries. Most respondents worked in Poland (10/62; 16.1%), the Netherlands (9/62; 14.5%) and Austria (6/62; 9.7%). Respondents were mostly affiliated to larger, regional or teaching (25/62; 40.3%) and university hospitals (24/62; 38.7%). Additionally, eight informants contributed to country case studies' semi-structured interviews. | Methods and Results |
| **Data collection** | | | | |
| 17 | Interview guide | Were questions, prompts, and guides provided by the authors? Was it pilot tested? | The online survey was developed, tested and administered in English, in a mobile phone-friendly format. Five cognitive pretesting sessions took place between 23 to 25 November 2020 and involved six testers from five countries, one of whom was a native-English speaker. Survey results sequentially | Methods |

| # | Item | Question/description | Answer | Section |
|---|---|---|---|---|
| | | | informed the design and focus of country case semi-structured interviews and the briefs, shared with informants prior to interviews. | |
| 18 | Repeat interviews | Were repeat interviews carried out? If yes, how many | No. However, some interview informants also participated to the other stages of the research, such as webinars and the online survey. | Methods |
| 19 | Audio/visual recording | Did the research use audio or visual recording to collect the data? | Interviews and webinars were audio recorded with the agreement of participants. | Methods |
| 20 | Field notes | Were field notes made during and/or after the interview or focus group? | All interviews were transcribed verbatim to follow. Webinar recordings were reviewed and detailed notes taken. | Methods |
| 21 | Duration | What was the duration of the interviews or focus groups? | Interviews lasted on average 30 minutes. Webinars lasted 60 and 90 minutes, respectively. | Methods |
| 22 | Data saturation | Was data saturation discussed? | Regular research team meetings were organized to discuss progress and recurrent themes. | Methods |
| 23 | Transcripts returned | Were transcripts returned to participants for comment and/or correction? | No. Transcripts were provided when requested by participants for their recordkeeping purposes. | Methods |
| **Domain 3: analysis and findings** | | | | |
| **Data analysis** | | | | |
| 24 | Number of data coders | How many data coders coded the data? | Data analysis was conducted by the first author (DI) and reviewed by co-authors (PG, NK, DK). | Methods |
| 25 | Description of the coding tree | Did authors provide a description of the coding tree? | No, the detailed data coding tree can be shared following a reasonable request but was not included in the paper manuscript nor its appendices. | NA |
| 26 | Derivation of themes | Were themes identified in advance or derived from the data? | Interview themes were derived from the online survey results. These results sequentially informed the design and focus of country case semi-structured interview guides. | Methods |
| 27 | Software | What software, if applicable, was used to manage the data? | An Excel tool was developed. | Methods |

| # | Item | Question/description | Answer | Section |
|---|---|---|---|---|
| 28 | Participant checking | Did participants provide feedback on the findings | Preliminary online survey and case study interview results were presented and discussed in subsequent webinars. | Methods |
| **Reporting** | | | | |
| 29 | Quotations pr-esented | Were participant quotations presented to illustrate the themes/findings? Was each quotation identified? e.g. participant number | Yes, participant quotes were used in the paper, to illustrate findings. Each quotation was assigned to a randomised and anonymised informant code (number). | Results |
| 30 | Data and findings consistent | Was there consistency between the data presented and the findings? | Findings are presented in the approach of the research questions and appropriate methodology used. | Introduction, Methods and Results |
| 31 | Clarity of major themes | Were major themes clearly presented in the findings? | Major themes have been presented in the results. | Results |
| 32 | Clarity of minor themes | Is there a description of diverse cases or discussion of minor themes? | Manuscript present both research findings on major and minor themes investigated in the study. These are also discussed in the Discussion section. | Results and Discussion |

[a] Tong A, Sainsbury P, Craig J. Consolidated criteria for reporting qualitative research (COREQ): a 32-item checklist for interviews and focus groups. Int J Qual Health Care 2007;19:349–57.
