# Supplementary material for: Data-Driven Collaboration between Hospitals and Other Healthcare Organisations in Europe During the COVID-19 Pandemic: An Explanatory Sequential Mixed-Methods Study among Mid-Level Hospital Managers
Source: Int J Integr Care. 2023 Jun 16;23(2):28. doi: 10.5334/ijic.6990 (PMC10275210; doi:10.5334/ijic.6990)
Supplement: Appendix 2. — Survey cognitive testing informants. [file ijic-23-2-6990-s2.pdf]

Appendix 2: Survey cognitive testing informants

Informant ID	Country	Type of (healthcare) organisation	Cognitive testing date
CT#1<br>CT#2	Republic of Moldova	Private for-profit hospital	23.11.2020
CT#3	Latvia	Academic public hospital	23.11.2020
CT#4	The Netherlands	Regional private non-for-profit hospital	24.11.2020
CT#5	Portugal	Regional public hospital	25.11.2020
CT#6	Ireland	Central government	25.11.2020
