# Supplementary material for: Data-Driven Collaboration between Hospitals and Other Healthcare Organisations in Europe During the COVID-19 Pandemic: An Explanatory Sequential Mixed-Methods Study among Mid-Level Hospital Managers
Source: Int J Integr Care. 2023 Jun 16;23(2):28. doi: 10.5334/ijic.6990 (PMC10275210; doi:10.5334/ijic.6990)
Supplement: Appendix 3. — Online survey (mobile version). [file ijic-23-2-6990-s3.pdf]

Appendix 3: Online survey (mobile version)

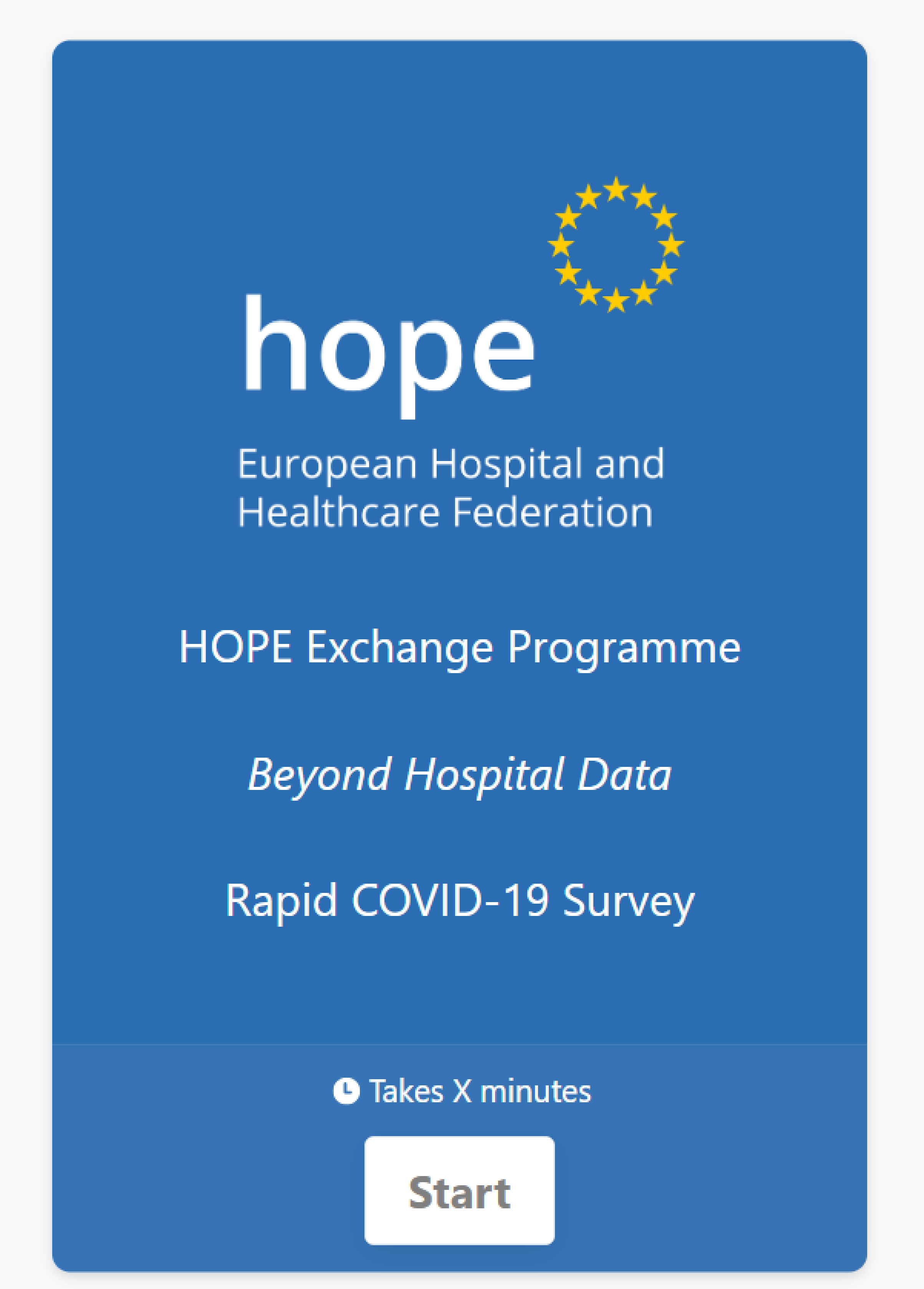

First of all, thank you for your invaluable front-line work!

We know you're super busy so we'll keep it super short.

This is a **one-minute** survey on COVID-related data integration between your hospital and other health care organisations.

One minute? Let's go!

Powered by **Typeform**

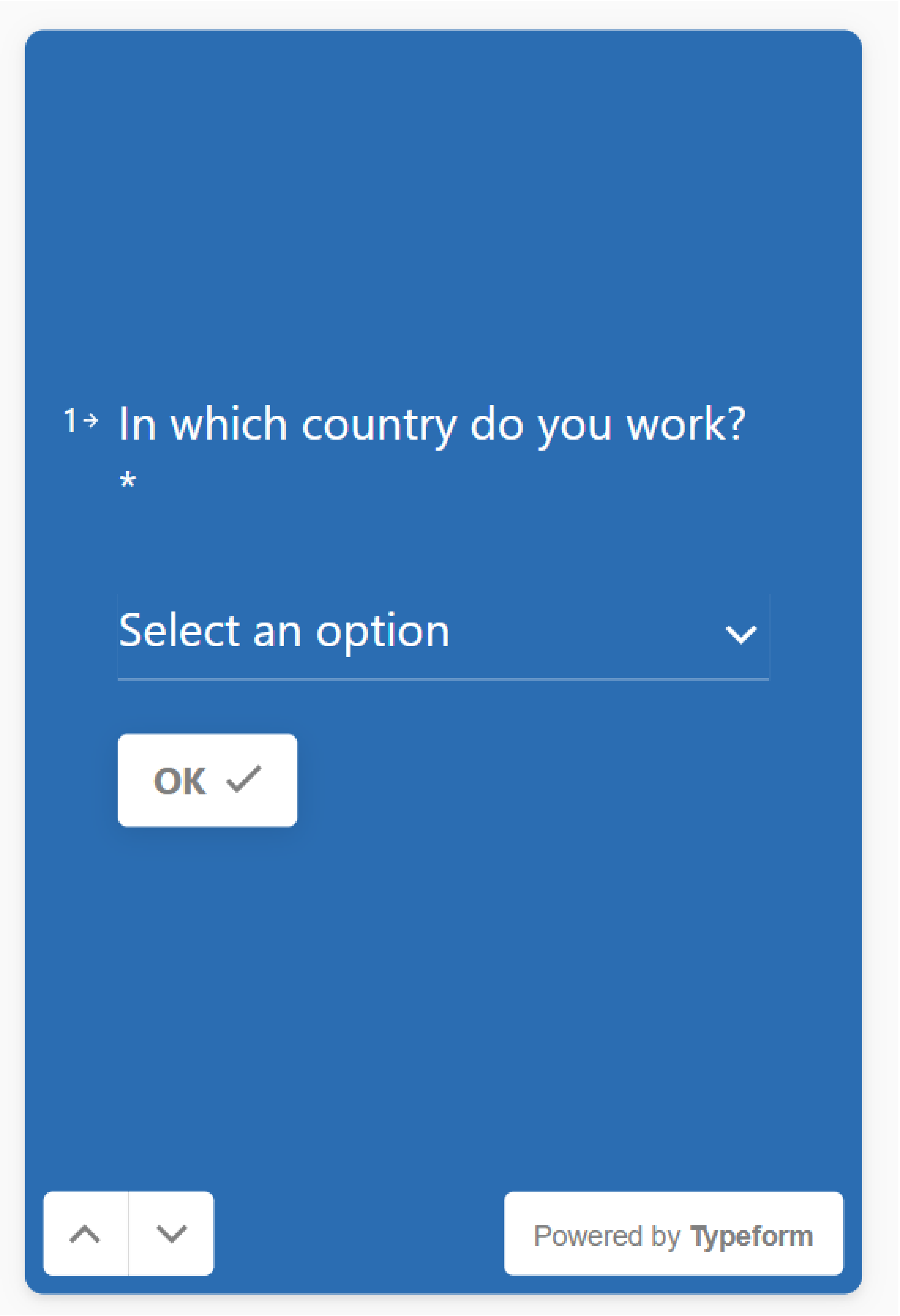
1→ In which country do you work? *
Select an option
OK
Powered by Typeform

2→ Do you work in a hospital that treats COVID-19 patients? *

Y Yes

N No

Powered by Typeform

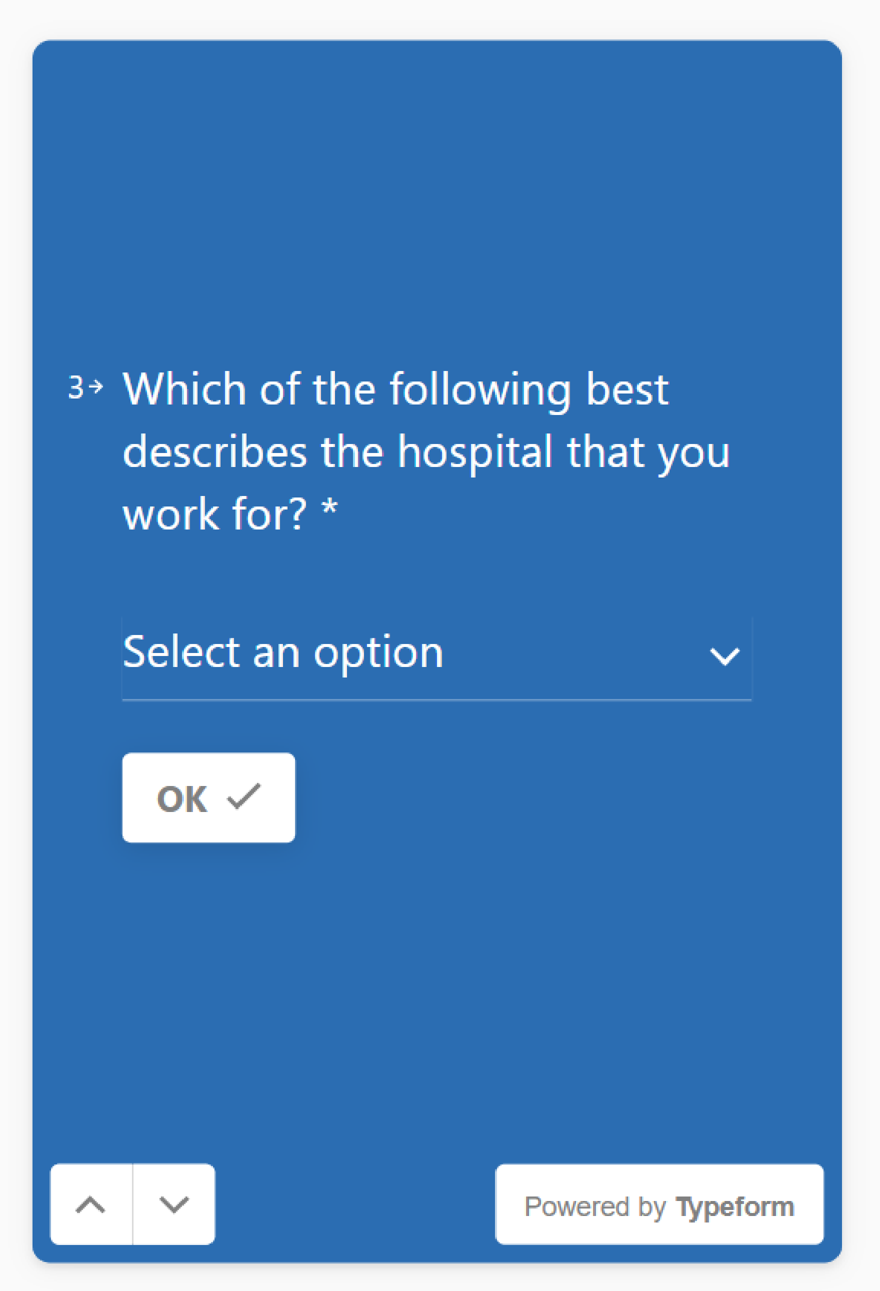
3→ Which of the following best describes the hospital that you work for? *

Select an option

OK ✓

Powered by Typeform

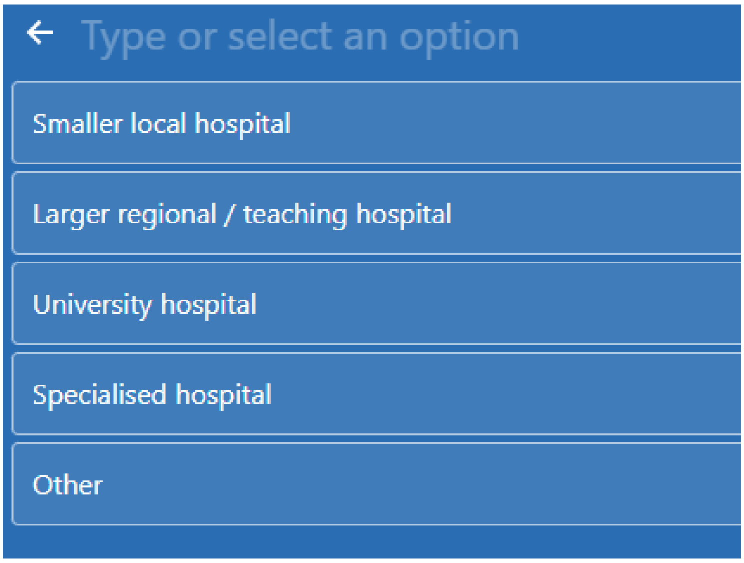
Type or select an option

- Smaller local hospital
- Larger regional / teaching hospital
- University hospital
- Specialised hospital
- Other

4→ Which of the following images best describes your hospital in relation to other healthcare organisations when it comes to exchanging data on COVID-19? *

This includes COVID-19 patients' clinical data but also data on relevant resources (e.g., PPE, respirators, qualified staff).

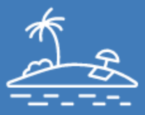

A An isolated island

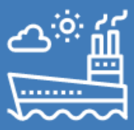

B An island connected by a ferry boat

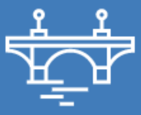

C An island connected by a bridge

OK ✓

Powered by Typeform

5 → Collaboration with other healthcare organisations during the COVID-19 pandemic has... *

1 → Decreased
3 → Stayed the same
5 → Increased

1	2	3	4	5

OK ✓

Powered by **Typeform**

6→ To what extent does your hospital exchange COVID-related data with **public health institutions** and their data systems?

This includes COVID-19 patients' clinical data but also data on relevant resources (e.g., PPE, respirators, qualified staff).
Note: Skip this question if not applicable.

1→Never
3→Ad-hoc
5→Daily/Real-time

1	2	3	4	5

Powered by **Typeform**

7→ To what extent does your hospital exchange COVID-related data with **long-term care institutions** and their data systems?

This includes COVID-19 patients' clinical data but also data on relevant resources (e.g., PPE, respirators, qualified staff).
Note: Skip this question if not applicable.

1 → Never
3 → Ad-hoc
5 → Daily/Real-time

1	2	3	4	5

Powered by **Typeform**

8→ To what extent does your hospital exchange COVID-related data with **primary care providers** and their data systems?

This includes COVID-19 patients' clinical data but also data on relevant resources (e.g., PPE, respirators, qualified staff).
Note: Skip this question if not applicable.

1→Never
3→Ad-hoc
5→Daily/Real-time

1	2	3	4	5

Powered by **Typeform**

9→ To what extent does your hospital exchange COVID-related data with **other hospitals** and their data systems?

This includes COVID-19 patients' clinical data but also data on relevant resources (e.g., PPE, respirators, qualified staff).
Note: Skip this question if not applicable.

1→Never
3→Ad-hoc
5→Daily/Real-time

1	2	3	4	5

Powered by **Typeform**

10→ Please elaborate on lessons learnt with the exchange of COVID-19 related data.

This can include new data exchange and linkage initiatives and possibilities, enablers and/or barriers to data exchange or anything else you find worthwhile to share with us.

Type your answer here...

That's all. Finish and submit!

Powered by **Typeform**

Create a typeform

Thank you!

Please, save the date for our Agora "Beyond Hospital Data" webinar on February 26th at 11am (CET)!

Until then, keep up the great work and stay safe.

Yours, HOPE
