# Supplementary material for: Data-Driven Collaboration between Hospitals and Other Healthcare Organisations in Europe During the COVID-19 Pandemic: An Explanatory Sequential Mixed-Methods Study among Mid-Level Hospital Managers
Source: Int J Integr Care. 2023 Jun 16;23(2):28. doi: 10.5334/ijic.6990 (PMC10275210; doi:10.5334/ijic.6990)
Supplement: Appendix 4. — Case study interview invitation. [file ijic-23-2-6990-s4.pdf]

Appendix 4: Case study interview invitation

HealthPros
Innovative Training Network for Healthcare Performance Intelligence Professionals

hope
European Hospital and Healthcare Federation

Beyond Hospital Data:
COVID-related data integration between hospitals and other health care organisations

Interview invitation (April 2021)

Dear HOPE Exchange Programme Participant, Local Host and / or National Coordinator,

Thank you for considering participating in our study exploring **COVID-related data integration between hospitals and other health care organisations -** from the perspective of managers working in hospitals. This document will briefly explain stages of this study, the work done so far, next steps and your potential role.

The study started in January with a **survey** among HOPE Exchange Programme participants (from 2019 onwards), local hosts and national coordinators. The results confirmed that hospitals are not *isolated islands* and that the COVID-19 pandemic did accelerate collaboration and data exchange between hospitals and other healthcare organisations – mainly with public health institutions, long-term care facilities, primary care providers and other hospitals. Equally important, the survey provided us with a number of short *testimonials* of your experiences and lessons learned during the pandemic. Results of the rapid survey are available here.

Next phase was the HOPE "Beyond Hospital Data" **webinar**, which took place in late February, where we presented survey results and discussed experiences in COVID-related data exchange from several European countries. Again, we were not surprised to find that discussing your individual experiences added a lot of depth and context to our collective understanding of what and how changed because of COVID-19, as well as how this can be sustained. Webinar recording is available here.

Now, for the third phase of this work, we look forward to **interviewing** you – as an individual involved with hospital managerial work during the COVID-19 pandemic. We would like to discuss your experience in working with COVID-19 data and exchanging it with other health care organisations in your country (and beyond). This interview is expected to last 60 minutes and, with your agreement, it will be audio recorded for the purpose of detailed notetaking.

If you are interested to participate, please email our interview coordinator, Damir Ivanković, before April 20th.

Finally, on June 11th, all participants and the HOPE community will be brought together in a **workshop** where we will present back the interview findings. Participants will also be given the opportunity to directly exchange experiences and lessons learned.

This work will provide us with a better understanding of what is happening in different hospitals around Europe in order to support you and the whole HOPE community in learning and improving.

Thank you in advance for your time and insights!

With appreciation, on behalf of the full study team,

Interview coordinator
Damir Ivanković
HealthPros PhD Fellow
d.ivankovic@amsterdamumc.nl

HOPE Co-ordinator
Pascal Garel
Chief Executive
sg@hope.be

HealthPros Co-ordinator
Niek Klazinga
Professor of Health Service Research
n.s.klazinga@amsterdamumc.nl

Disclaimer: This project has received funding from the European Union's Horizon 2020 research and innovation programme under grant agreement No. 765141. This document reflects only the author's views and the European Commission is not responsible for any use that may be made of the information it contains.
