# Supplementary material for: Data-Driven Collaboration between Hospitals and Other Healthcare Organisations in Europe During the COVID-19 Pandemic: An Explanatory Sequential Mixed-Methods Study among Mid-Level Hospital Managers
Source: Int J Integr Care. 2023 Jun 16;23(2):28. doi: 10.5334/ijic.6990 (PMC10275210; doi:10.5334/ijic.6990)
Supplement: Appendix 5. — Case study interview informants. [file ijic-23-2-6990-s5.pdf]

Appendix 5: Case study interview informants

Informant ID	Country	Type of (healthcare) organisation	Interview date
Case study informant #1	The Netherlands	Private not-for profit hospital	21.4.2021
Case study informant #2	Poland	Regional public hospital	29.4.2021
Case study informant #3	Poland	Local public hospital	3.5.2021
Case study informant #4	France	Public university hospital	4.5.2021
Case study informant #5	Belgium	National hospital association	20.5.2021
Case study informant #6	Finland	Association of local and regional health authorities	1.6.2021
Case study informant #7	Republic of Moldova	Private for-profit hospital	21.6.2021
Case study informant #8	Ireland	Private for-profit hospital	22.6.2021
